# Thin‐film CdTe detector for microdosimetric study of radiation dose enhancement at gold‐tissue interface

**DOI:** 10.1120/jacmp.v17i5.6339

**Published:** 2016-09-08

**Authors:** Nava Raj Paudel, Diana Shvydka, E. Ishmael Parsai

**Affiliations:** ^1^ Department of Radiation Oncology The University of Toledo Medical Center Toledo OH USA; ^2^ Department of Radiation Oncology University of Arkansas for Medical Sciences Little Rock AR USA

**Keywords:** Monte Carlo study, Cadmium Telluride (CdTe) detector, interface microdosimetry, interface effect, HDR brachytherapy

## Abstract

Presence of interfaces between high and low atomic number (Z) materials, often encountered in diagnostic imaging and radiation therapy, leads to radiation dose perturbation. It is characterized by a very narrow region of sharp dose enhancement at the interface. A rapid falloff of dose enhancement over a very short distance from the interface makes the experimental dosimetry nontrivial. We use an in‐house‐built inexpensive thin‐film Cadmium Telluride (CdTe) photodetector to study this effect at the gold‐tissue interface and verify our experimental results with Monte Carlo (MC) modeling. Three‐micron thick thin‐film CdTe photodetectors were fabricated in our lab. One‐, ten‐ or one hundred‐micron thick gold foils placed in a tissue‐equivalent‐phantom were irradiated with a clinical Ir‐192 high‐dose‐rate (HDR) source and current measured with a CdTe detector in each case was compared with the current measured for all uniform tissue‐equivalent phantom. Percentage signal enhancement (PSE) due to each gold foil was then compared against MC modeled percentage dose enhancement (PDE), obtained from the geometry mimicking the experimental setup. The experimental PSEs due to 1, 10, and 100 μm thick gold foils at the closest measured distance of 12.5 μm from the interface were 42.6±10.8, 137.0±11.9, and 203.0±15.4, respectively. The corresponding MC modeled PDEs were 38.1±1., 164±1, and 249±1, respectively. The experimental and MC modeled values showed a closer agreement at the larger distances from the interface. The dose enhancement in the vicinity of gold‐tissue interface was successfully measured using an in‐house‐built, high‐resolution CdTe‐based photodetector and validated with MC simulations. A close agreement between experimental and the MC modeled results shows that CdTe detector can be utilized for mapping interface dose distribution encountered in the application of ionizing radiation.

PACS number(s): 29.40.Wk, 73.50.Pz, 87.53.Jw, 87.55.K‐

## I. INTRODUCTION

Dose perturbation at the interface between high‐and low‐Z materials has been a subject of investigation over the past several decades. The interplay between problem details such as difference in Z, radiation energy, and interface geometry lead to different amounts of primary beam attenuation and scatter, calling for a dosimetric evaluation in each specific case. While attempts have been made for a comprehensive coverage of extended energy range,^(1)^ there is no universal method for accurate quantitative description of the interface dosimetry. However, a high‐resolution microdosimetry approach should be employed for such studies since most of the dose perturbation effects occur over a very short (∼1 mm) distance from the interface.

The traditional methodology of microdosimetry goes back to the work by H. H. Rossi^(2)^ and the measurement of energy concentration using Rossi counters and single wire proportional counters.^(3)^ However, unsuitability of Rossi detectors in mapping interface effect in heterogeneous materials^(4)^ prompted the development of alternative high spatial resolution methodologies. Together with MC simulations,^5^, ^6^, ^7^, ^8^ various detectors such as thin window parallel plate ion chambers^9^, ^10^ and thermally stimulates exo‐electron (TSEE) detectors^1^, ^11^, ^12^, ^13^ have been utilized in the experimental microdosimetric studies of the interfaces. Semiconductor detectors, well known for their high spatial resolution, radiation absorption efficiency, stability, and wide dynamic range, have also been extensively used for dosimetric purposes. Thin solid‐state detectors offering a real‐time data acquisition at low or no voltage external bias^(14)^ are quite convenient for relative dosimetric studies. Silicon‐on‐insulator (SOI) p‐n junction diode as thin as 2 μm has been explored for microdosimetric applications.^15^, ^16^ While radiation properties close to tissue make silicon detectors attractive for measurements in biological and medical systems, their poor detection efficiency and nonlinear energy response^(14)^ complicate the data interpretation, especially for the photon sources with wide spectra. A clinical HDR brachytherapy Ir‐192 source falls in this category, with the energy spectrum ranging from 8.91 keV to 1.38 MeV.^17^, ^18^

CdTe, on the other hand, is more sensitive and suitable for dosimetric applications in both photoelectric and Compton energy range due to its higher average Z and electron density. The most common CdTe‐based photodetector is a thin film p‐n junction diode, formed by p‐type (CdTe) and n‐type (Cadmium Sulfide (CdS)) materials, which can be operated without any external bias (photovoltaic mode). It offers a linear energy response and does not require cooling for the measurement. Application possibility of CdTe detectors in megavoltage, as well as diagnostic X‐ray imaging, has been explored.^19^, ^20^ Recent developments in thin‐film photovoltaic applications have made thin low‐cost, high‐quality CdTe devices the potentially viable choice for microdosimetry.

Here we investigate the suitability of an in‐house‐built, thin‐film CdTe‐based photodetector for relative microdosimetry at the gold‐tissue interface under a clinical Ir‐192 irradiation. In this problem, the rapid dose falloff over a submillimeter distance requires a high spatial resolution instrument for the interface dosimetry. For simplicity, we approach the problem in one‐dimensional geometry, and verify the experimental results with MC simulations obtained from the geometry replicating the experimental setup.

## II. MATERIALS AND METHODS

### A. CdTe detector fabrication and its operation

A number of thin‐film CdTe diode photodetectors (sensitive volume dimensions: 3 μm thickness and $0.95\;{\text{cm}}^{2}$ contact area) were prepared in our lab using radiofrequency‐sputtering following a typical CdTe‐based photovoltaic device fabrication procedure.^(21)^ TEC‐15 glass from Pilkington, covered with transparent conducting oxide (TCO), was used as the substrate. This soda‐lime glass is 3 mm thick. Fluorine‐doped Tin Oxide (SnO2:F) TCO layer, having 15 Ohm per square (Ω/□) sheet resistance, served as the bottom electrode. A thin layer of CdS was deposited on top of the TCO, followed by a layer of CdTe. After the deposition of the semiconductor layers, the device was subjected to a high‐temperature annealing step in the presence of cadmium chloride (${\text{CdCl}}_{2}$) flux agent. This process passivates grain boundaries and drastically improves the device performance. A top metal electrode (copper‐gold double layer), defining the area of the device was evaporated and heat‐diffused as a finishing step.

**Figure 1:**
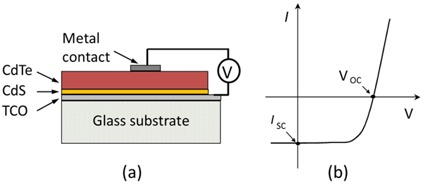
A sketch (a) of a CdTe‐based photodetector on a glass substrate (thicknesses are not to the scale), showing the connections for an output measurement. Characteristic I‐V curve (b) of a CdTe‐based photodetector, with $V_{\text{OC}}$ and $I_{\text{SC}}$ points marked.

CdTe was at least an order of magnitude thicker than any other layer. The structure of a CdTe detector (not to the scale) is presented in Fig. 1(a).

A CdTe device is used in the photovoltaic mode, where electron‐hole pairs created by incoming and secondary radiation in the sensitive volume of the diode are separated by a built‐in electric field and collected at two terminals. An output is obtained through the top metal layer and the bottom TCO contact, as shown in Fig. 1(a). A characteristic current‐voltage (I‐V) curve of a CdTe detector is shown in Fig. 1(b), where $V_{\text{OC}}$ is the open circuit voltage and $I_{\text{SC}}$ is the short circuit current.

### B. Experimental procedure

High‐purity gold foils (99.9% pure, Goodfellow Corporation, http://www.goodfellow.com/) of 1.0±0.0 μm, 10.0±1.0 μm, and 100.0±0.0 μm thicknesses (mean ± standard deviation, measured with a digital caliper) were used in this experiment. One‐micron‐thick foil was the thinnest foil available commercially, while 100 μm‐thick foil was chosen for its thickness close to the range of 382 keV secondary electrons, ejected by photons from the Ir‐192 HDR source (average energy ∼382 keV).^(22)^

**Figure 2:**
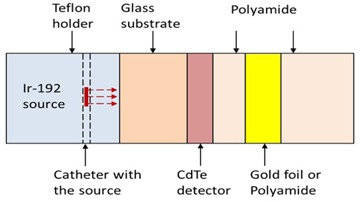
Geometry of the experimental setup, also replicated with MC model. Dashed arrows show the direction of photons emitted by the Ir‐192 HDR source.

Polyamide (Kapton) sheets (Dupont, www.dupont.com), with measured mean ± standard deviation thicknesses of 12.5±0.0, 27.5±0.0, 55.0±0.0, and 127.0±0.0 μm and density ($1.42\;g/{\text{cm}}^{3}$) slightly higher than that of tissue, were used to vary the distance between the detector and the gold foil. The distance between the source and the detector was fixed (4.5 mm) for each measurement, while the distance between the detector and the gold foil was varied by placing different number and/or thicknesses of polyamide sheets in between them. Thus, the detector, Polyamide sheets, and the gold foil were placed parallel to each other and irradiated with a single dwell position of the clinical Ir‐192 HDR source (VariSource IX Afterloader, Varian Medical Systems, Palo Alto, CA) placed in a catheter fixed in the middle of a Teflon holder just underneath the middle of the detector. Radiation from the source was incident perpendicular to the center of detector surface. The experimental setup is schematically shown in Fig. 2, where “Gold foil or Polyamide” layer identifies the position of the gold foil. Polyamide was placed in this layer for the reference measurement, while gold foil was placed to measure its effect on dose enhancement. As evident from Fig. 2, CdTe detector sampled the region of dose enhancement due to secondary electrons backscattered from the gold foil. The effect of backscattered radiation was measured in this study for the experimental simplicity. Two pogo‐pins attached to the bottom and top layers of the detector were connected to a Keithley 2600A source meter (Keithley Instruments B.V., Gorinchem, The Netherlands) to measure the device output. Since CdTe is sensitive to visible light, the detector was covered with a black cloth and the measurements were carried in a dark room in order to minimize the effect of light.

**Figure 3:**
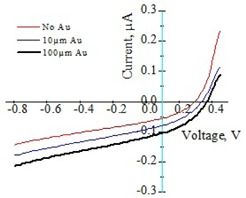
Characteristic CdTe photodetector I‐V curves acquired under Ir‐192 source irradiation without gold, and with 10.0±1.0 and 100±0.0 μm‐thick gold foils.

Electrical connection for each setup was verified by obtaining a full I‐V curve (Fig. 3) using the source meter while irradiating the system with the Ir‐192 source. Since the current generated in the sensitive volume of the detector is proportional to the energy deposited, $I_{\text{SC}}$ was used to evaluate the signal enhancement. For each data point, 200 values of $I_{\text{SC}}$ were collected over a period of 10 s at zero external bias while irradiating the detector in the presence and absence of gold foils, and the corresponding data collected were averaged. The measurements were repeated for distances between gold foil and the detector varying from 12.5 to 500 μm, 12.5 to 1000 μm, and 12.5 to 1250 μm for 1, 10, and 100 μm foil thicknesses, respectively. PSE was defined in terms of $I_{\text{SC}}$ as follows:

(1) $$PSE=\frac{I_{\text{gold}}-I}{I}\times 100\%$$

Here, $I_{\text{gold}}$ and *I* are $I_{\text{SC}}$ readings when the ‘gold foil or polyamide’ layer (Fig. 2) contained gold foil and polyamide, respectively.

### C. Monte Carlo simulation

The experimental geometry shown in Fig. 2 was explicitly replicated using radiation transport package MCNP5.^(23)^ Energy deposited in the sensitive volume of the detector was obtained with the energy deposition tally *F8, which records contribution by each source particle and its secondary particles (unit: MeV per source particle), and converted into dose. The PDE due to the gold foils was then defined as follows:

(2) $$PDE=\frac{D_{\text{gold}}-D}{D}\times 100\%$$

Here, $D_{\text{gold}}$ and *D* are dose deposited in sensitive volume of the CdTe detector when the ‘gold foil or polyamide’ layer (Fig. 2) contained gold foil and polyamide, respectively.

The energy deposition leads to generation of electron‐hole pairs (career ionization energy of polycrystalline CdTe∼5 eV).^(24)^ While a fraction of the generated charge carriers are lost due to recombination, the rest contribute to the measured short‐circuit current. Since both current generated and dose deposited in the sensitive volume of the detector are proportional to the energy deposited, our experimental results were validated by comparing the enhancement in experimentally measured current generation against the MC modeled enhancement in dose deposition.

For all MC simulations, the number of histories ($N=10^{7}-10^{8}$) was adjusted to keep the errors in energy deposition tallies below 1%, with cutoff energies set to 1 keV for both photons and electrons. Cell sizes used in the simulation were determined by the actual size of the devices used, such as detector, polyamide, and gold foils. MC modeling package, MCNP5, used in our study relies on condensed history algorithm for electron simulations, resulting in significant increase in the calculation speed.^(25)^ This did not cause any problem for the dimensions and photon energies used in this study.

## III. RESULTS & DISCUSSION

Our previous MC simulations^26^, ^27^ have shown an almost symmetric pattern of dose enhancement about the gold‐tissue interface for perpendicular incidence of radiation from an Ir‐192 source. The symmetry arises from almost equal contribution of forward directed and back scattered secondary electrons from the interface.

A set of typical I‐V curves obtained for verification of the experimental setup in the present study are shown in Fig. 3. These curves were obtained by irradiating the CdTe device/Polyamide sheet assembly with an Ir‐192 HDR source in the presence and absence of 10.0±1.0 and 100±0.0 μm‐thick gold foils. Shift of the curve along the negative y‐axis, corresponding to $I_{\text{SC}}$, indicates the signal enhancement due to the foils.

Both experimental and MC modeled results are summarized in 4, 5, 6 for all three thicknesses of the gold foils. The error bars for experimental data represent the standard deviations (SD) for 200 values collected for each data point. Error bars for MC modeled data, represented by the size of the filled squares in the corresponding graphs, are significantly smaller than the experimental error bars.

As evident from 4, 6, the signal enhancement due to thicker gold foils extended over a longer distance from the interface with the tissue‐equivalent material. Experimental signal enhancements due to the gold foils of all three thicknesses closely followed the dose enhancements obtained from MC results except for the higher differences at the points closest (12.5 μm) to the gold foil. Higher differences were also obtained at other distances for the 100 μm‐thick gold foil. The measured thicknesses of gold and polyamide have no or negligible variation in magnitude. For 10±1 μm thick gold, the uncertainty in thickness resulted in a variation in dose enhancement only by ±7%, while the difference between the experimental and simulated mean values at 12.5 μm distance was 27%. However, the added uncertainty due to the uncertainty in thickness of 10 μm foil provided a wider window in the error margin. Other thicknesses of gold and Polyamide have zero measured variation. Hence, the difference between the experimental and simulated results cannot be attributed to the thickness variation. Slight variation in detector thickness would not contribute to relative current enhancement. One possible factor contributing to the discrepancy between the measured and the simulated results is an electric field due to the radiation‐induced charge accumulation within the glass substrate. Electrostatic forces arising from the electric field may affect the distribution of secondary electrons backscattered from the foil. The electric field, known with multiple applications,^(28)^ has not been accounted for in the MC model. It would have the strongest effect on the data points obtained at the shortest distances from the gold‐polyamide interface. Additionally, experimental setup uncertainties (e.g., thin air pockets between multiple polyamide sheets) not included in the standard deviations, depicted by error bars for measured values in 4, 6, may contribute to the discrepancies between modeled and experimental dataset. These would add up to be most evident at the distances furthest from the foil, as illustrated by 5, 6 for thicker foils, where data points extend to the larger distances.

**Figure 4:**
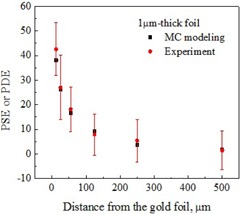
Measured PSE and MC modeled PDE near the 1.0±0.0 μm‐thick gold foil interface with the tissue‐mimicking polyamide.

**Figure 5:**
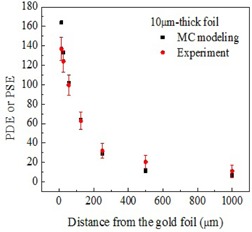
Measured PSE and MC modeled PDE near the 10.0±1.0 μm‐thick gold foil interface with the tissue‐mimicking polyamide.

**Figure 6:**
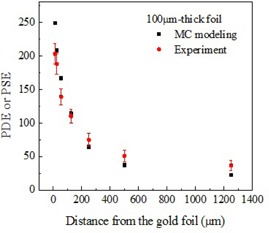
Measured PSE and MC modeled PDE near the 100.0±0.0 μm‐thick gold foil interface with the tissue‐mimicking polyamide.

As the distance between the gold foil and the detector decreases due to the presence of thinner dielectric medium (polyamide) between them, electric field distribution may also change. This can probably be remedied by using detectors fabricated on a different substrate, such as thin polyamide or metal. Such detector configurations are under development in our group.

Among the published studies, a recent study^(29)^ utilizing Ir‐192 source and a thin (5 μm) window parallel plate ion chamber most closely resembles the conditions of our experiment. Dose enhancement of 3.44 (upstream value) calculated with MC simulations for 0.1 mm thick Au foil in the study is higher than our maximum value of 2.5 (downstream) for the same foil thickness (see Fig. 4). We attribute the difference to the noticeable differences in simulation geometries. While simulations in the study by Zhang and Das^(29)^ were conducted for a uniform water medium, our simulation closely replicated the experimental setup, which included multiple interfaces (e.g., glass substrate, CdTe layer) before the Au/polyamide interface. A MC simulation of our problem geometry with glass, polyamide, and CdTe materials replaced with water resulted in a PDE of ~ 3 (scored in 3 μm water layer) at 12.5 μm distance from the 100 μm‐thick gold foil.

Other published microdosimetry experiments performed at the interface between high‐ and low‐Z materials were conducted at significantly different energies. A study by Das and Chopra^(10)^ has shown a dose enhancement factor of about 15 at lead‐polystyrene interface under 200 kVp ($E_{\text{avg}}∼67\;\text{keV}$) beam using a thin window parallel plate ion chamber of 5 μm thickness. Another study by Regulla et al.^(13)^ has shown a dose enhancement factor of 114 at gold‐polymethyl methac‐rylate interface under 60 kVp ($E_{\text{avg}}∼20\;\text{keV}$) beam using a 0.1 μm‐thick TSEE detector. The average energies of the radiation sources used in those experiments are 6 to 19 times lower than in our experiment. Hence lower dose enhancements in our study, performed at higher average energy of 380 keV, are as expected. Measured dose enhancement also depends on the spatial resolution of the measuring device, leading to dose averaging.

## IV. CONCLUSIONS

Our in‐house‐built, thin‐film CdTe detector has been successfully validated as a high‐resolution instrument for the interface dosimetry with adequate accuracy. The rapid dose falloff extending over a distance of ∼1 mm from the gold‐tissue interface was mapped with the detector offering 3 μm spatial resolution. Dark current is limited under zero external bias, and output short circuit current readings are proportional to the dose deposited within the sensitive volume of the detector. Close agreement of measured signal enhancement with MC modeled dose enhancement proves the suitability of the proposed approach to microdosimetric applications.

The spatial resolution achieved with the inexpensive thin‐film CdTe semiconductor photodetector is not attainable with a typical clinical dosimeter. A technically confirmed method to measure radiation dose enhancement in a narrow high‐dose gradient region under HDR brachytherapy radiation source can be utilized to study interface effects in various situations utilizing ionizing radiation.

## ACKNOWLEDGMENTS

This work was partially supported by NRC faculty development grant NRC‐HQ‐12‐G‐38‐0042.

## COPYRIGHT

This work is licensed under a Creative Commons Attribution 3.0 Unported License.
